# Comprehensive Assessment of Acute Isolated or Prominent Dysarthria in the Emergency Department: A Neuro-Emergency Expert’s Experience beyond Stroke

**DOI:** 10.3390/brainsci12081047

**Published:** 2022-08-07

**Authors:** Soon-Ho Lee, Sang-Ook Ha, Jin-Hyouk Kim, Won-Seok Yang, Young-Sun Park, Tae-Jin Park

**Affiliations:** 1 Department of Emergency Medicine, Hallym University Sacred Heart Hospital, Hallym University Medical Center, Anyang 14068, Korea; 2 Department of Neurology, Hallym University Sacred Heart Hospital, Hallym University Medical Center, Anyang 14068, Korea; 3 Department of Emergency Medicine, National Medical Center, Seoul 03080, Korea

**Keywords:** emergency department, dysarthria, ischemic stroke

## Abstract

We investigated the clinical characteristics, neuroimaging findings, and final diagnosis of patients with acute isolated or prominent dysarthria who visited the emergency department (ED) between 1 January 2020 and 31 December 2021. Of 2028 patients aged ≥ 18 years with neurologic symptoms treated by a neuro-emergency expert, 75 with acute isolated or predominant dysarthria within 1 week were enrolled. Patients were categorized as having isolated dysarthria (*n* = 28, 37.3%) and prominent dysarthria (*n* = 47, 62.7%). The causes of stroke were acute ischemic stroke (AIS) (*n* = 37, 49.3%), transient ischemic attack (TIA) (*n* = 14, 18.7%), intracerebral hemorrhage (*n* = 1, 1.3%), and non-stroke causes (*n* = 23, 30.7%). The most common additional symptoms were gait disturbance or imbalance (*n* = 8, 15.4%) and dizziness (*n* = 3, 13.0%) in the stroke and non-stroke groups, respectively. The isolated dysarthria group had a higher rate of TIA (*n* = 7, 38.9%), single and small lesions (*n* = 10, 83.3%), and small-vessel occlusion in Trial of Org 101072 in acute stroke treatment (*n* = 8, 66.7%). Acute isolated or prominent dysarthria in the ED mostly presented as clinical symptoms of AIS, but other non-stroke and medical causes were not uncommon. In acute dysarthria with ischemic stroke, multiple territorial and small and single lesions are considered a cause.

## 1. Introduction

Acute dysarthria, a motor speech disorder in which speech process or production is impaired, is a neurological symptom encountered in the emergency department (ED). Acute dysarthria is common accompanied by other neurological symptoms, such as motor weakness, mainly in acute ischemic stroke at a rate of 26–56% [1,2,3]. However, it rarely occurs alone or with minor neurological symptoms in patients with acute ischemic stroke [4,5,6,7]. In the field of stroke, it has been called isolated or prominent dysarthria and is sometimes described as a special neurological syndrome, such as dysarthria-clumsy hand syndrome, dysarthria-facial paresis syndrome, and dysarthria-lingual palsy syndrome [8,9,10,11]. Pure dysarthria has been described as lacunar syndrome in internal capsule, corona radiata, small cortical, or subcortical lesions [10,12].

However, this isolated or prominent dysarthria is a challenging symptom for ED physicians because it is caused by not only acute ischemic stroke but also other conditions, such as multiple sclerosis [13]. To date, there are insufficient clinical data and comprehension of patients with isolated or prominent dysarthria in the ED environment. We investigated the clinical characteristics, neuroimaging findings, and final diagnosis of patients with acute isolated or prominent dysarthria who visited the ED. In addition, we hypothesized that there would be different clinical characteristics between isolated dysarthria and prominent dysarthria group in acute ischemic stroke or transient ischemic attack (TIA). 

## 2. Materials and Methods

### 2.1. Study Design and Patient Selection

This study was conducted at the ED of an urban academic tertiary-care hospital in South Korea. This 900-bed facility has a regional emergency medical center, as designated by the government, and receives approximately 75,000 ED visits annually. In our neuro-emergency medicine project [14], a neuro-emergency expert was responsible for the emergency care of patients with neurological symptoms. Through a systematic review of electronic medical records between 1 January 2020 and 31 December 2021, patients aged ≥18 years with neurologic symptoms treated by a neuro-emergency expert in the ED were screened. Among these patients, those with acute isolated or prominent dysarthria occurring within 1 week were enrolled. Patients with aphasia, altered mentality beyond deep drowsiness, definite motor weakness (The Modified Medical Research Council [MRC] grade ≤ 4), visual field defect, or National Institutes of Health Stroke Scale (NIHSS) score ≥ 5 were excluded. Neurological examination was assessed directly by a neuro-emergency expert at admission to ED. Patients who did not recognize the symptoms themselves but confirmed mild motor weakness (modified MRC grade 4+, 5−) through neurological examination were included in this study. 

Isolated dysarthria was defined as having only dysarthria and no neurological symptoms of other subjective or transient motor weakness, sensory changes, ataxia, gait disturbance, vertigo, or visual symptoms. Prominent dysarthria had other neurologic symptoms or systemic symptoms, such as fever or pain, but was defined as complaining of the main symptoms as dysarthria. NIHSS was investigated only in patients with acute ischemic stroke or TIA. All patients with TIA were received an NIHSS score of 0 with improved neurological symptoms at ED. 

This study was approved by the institutional review board of Hallym University Sacred Heart Hospital, which waived the requirement for informed consent (IRB no. 2022-07-002).

### 2.2. Data Collection

Data on patients’ baseline characteristics (age, sex, coexisting condition, antithrombotic agent, and time interval from onset to door), additional neurological or general symptoms, neuroimaging findings, NIHSS score, final diagnosis, and Trial of Org 101072 in Acute Stroke Treatment (TOAST) [15] were obtained and analyzed. When accompanied by multiple neurological or general symptoms, the symptom evaluated to be the most severe among these was analyzed as an additional symptom. 

### 2.3. Statistical Analyses

The normality of the data distributions was evaluated using the Kolmogorov–Smirnov test for the selection of appropriate parametric and non-parametric statistical methods. Categorical variables were analyzed using chi-square or Fisher’s exact tests and are expressed as total numbers (percentages). Continuous variables are expressed as medians (interquartile range) and were analyzed using the Mann–Whitney U test. For all comparisons, the tests were two-tailed, and between-group differences were considered significant at *p* < 0.05. Statistical Package for the Social Sciences for Windows ver. 18.0.0 (IBM, Armonk, NY, USA) was used for all analyses.

## 3. Results

**Figure 1:**
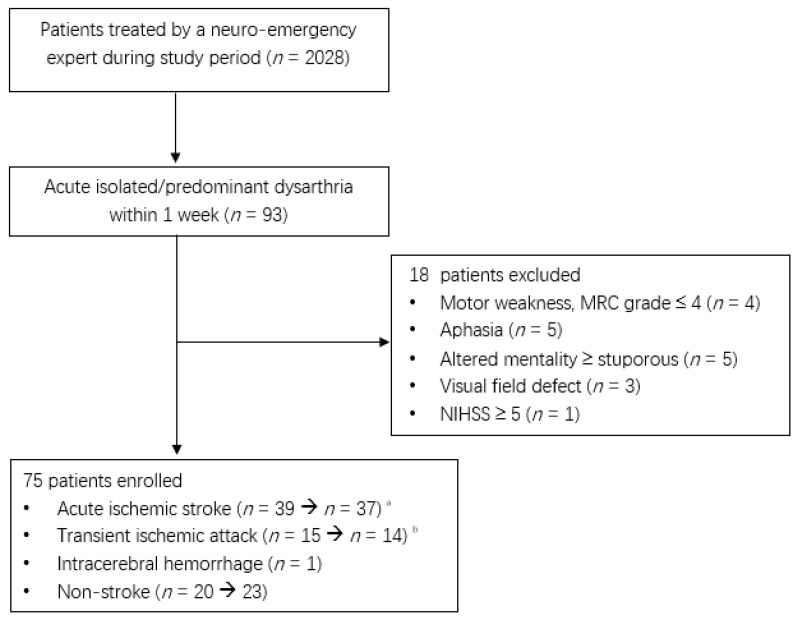
Flow diagram of enrolled patients during the study period (1 January 2020 to 31 December 2021). ^a^. Two patients were diagnosed with multiple sclerosis and multiple cranial neuropathies. ^b^. One patient’s diagnosis was changed to focal seizures.

Of the 2208 patients treated by a neuro-emergency expert, 75 patients, excluding 18 patients with acute isolated or predominant dysarthria within 1 week, were enrolled in the present study (Figure 1). Two patients with an initial acute ischemic stroke were diagnosed with multiple sclerosis and multiple cranial neuropathies.

As shown in Table 1, the study population was divided into isolated dysarthria (*n* = 28, 37.3%) and prominent dysarthria (*n* = 47, 62.7%) groups. We noted that the median onset-to-door time of the isolated dysarthria group was shorter than that of the prominent dysarthria group (689 min vs. 1283 min, *p* = 0.042). 

As shown in Table 2, the causes of stroke in acute isolated or prominent dysarthria were acute ischemic stroke (*n* = 37, 49.3%), TIA (*n* = 14, 18.7%) and intracerebral hemorrhage (*n* = 1, 1.3%). The number of patients with non-stroke causes was 23 (30.7%), including metabolic causes with hypoglycemia and uremia (*n* = 7, 9.3%), toxic causes (*n* = 6, 8.0%), brain tumor (*n* = 2, 2.7%), and Parkinson’s disease (*n* = 2, 2.7%). 

Table 3 shows various additional symptoms of patients with prominent dysarthria. The most common additional symptoms were gait disturbance or imbalance (*n* = 9, 12.0%), facial palsy (*n* = 8, 10.7%), and mild hemiplegia (*n* = 5, 6.7%) in order of frequency. This trend was similar to the stroke group, but dizziness was the most common additional symptom in the non-stroke group (*n* = 3, 13.0%).

**Figure 2:**
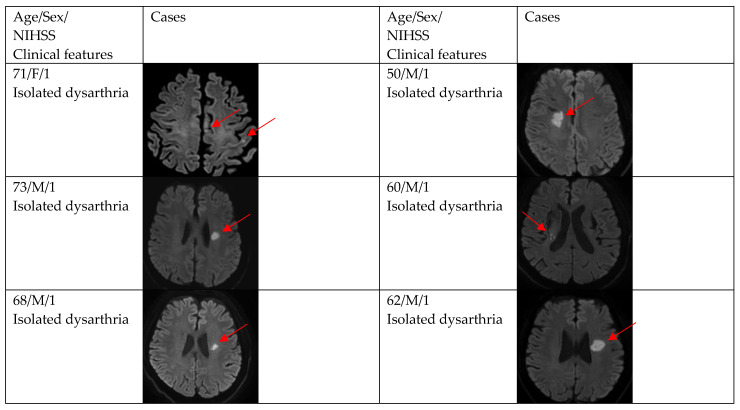

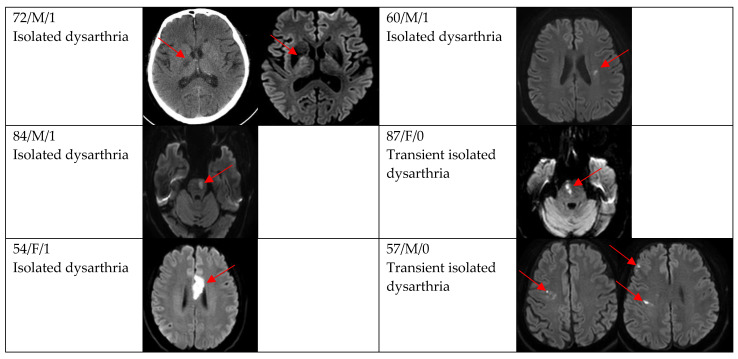
Clinical characteristics and neuroimaging findings of patients with ischemic stroke or TIA presenting with dysarthria only. Except for the bottom two cases, all showed a single lesion on DWI.

**Figure 3:**
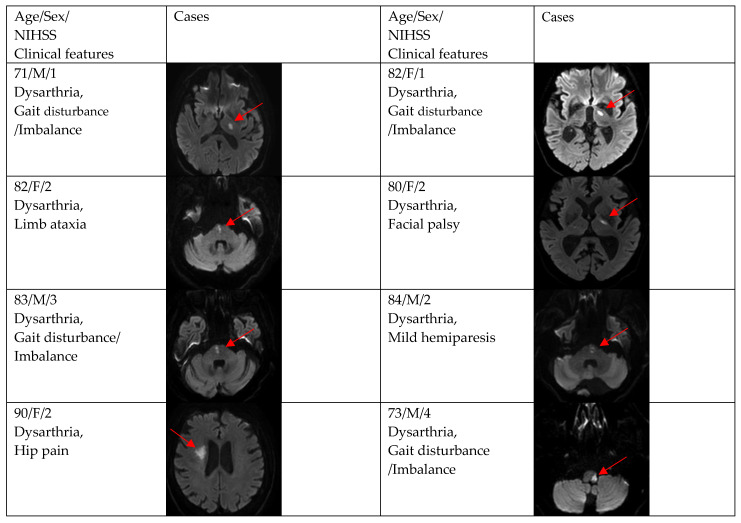

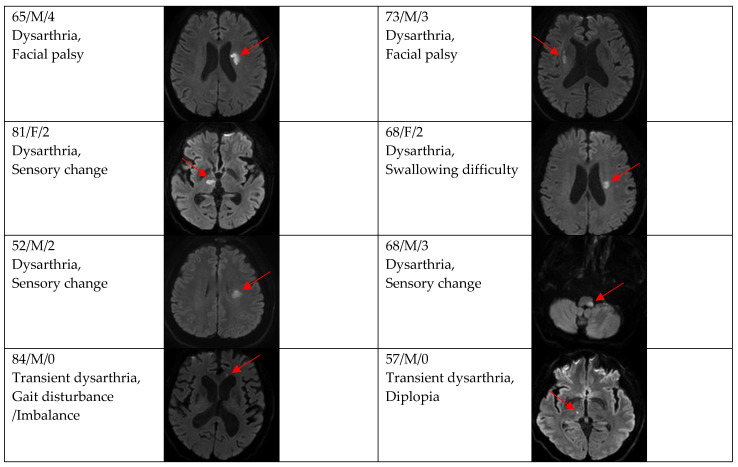
Clinical characteristics and neuroimaging findings of patients with ischemic stroke or TIA presenting with prominent dysarthria and small and single lesions.

**Figure 4:**
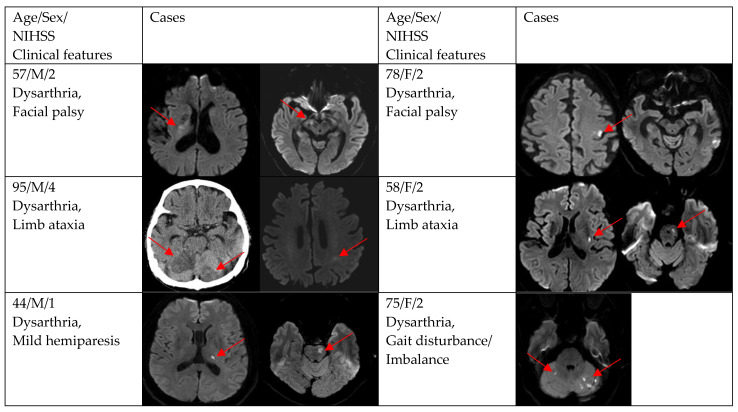

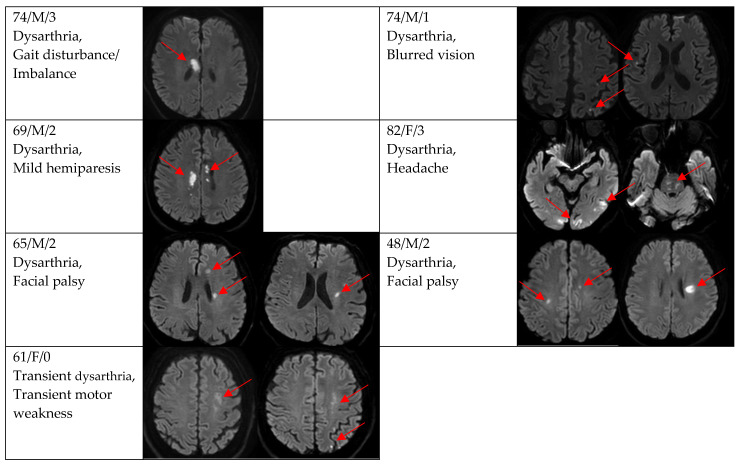
Clinical characteristics and neuroimaging findings of patients with ischemic stroke or TIA presenting with prominent dysarthria and multiple territorial lesions.

Figure 2, Figure 3 and Figure 4 show acute stroke lesions on diffusion-weighted imaging (DWI). In the isolated dysarthria group, 10 patients had small and single lesions, and 2 patients had multiple or territorial lesions on DWI. In the prominent dysarthria group, 16 patients had small and single lesions, and 13 patients had multiple or territorial lesions on DWI. 

In the sub-analysis of acute ischemic stroke or TIA, Table 4 shows the comparison between the isolated dysarthria group (*n* = 18, 35.3%) and the prominent dysarthria group (*n* = 33, 64.7%). The isolated dysarthria group had a higher proportion of TIA and lower proportion of DWI positivity than the prominent dysarthria group (*n* = 7, 38.9% vs. 7, 21.2%, *p* = 0.204, and *n* = 12, 66.7% vs. 29, 87.9%, *p* = 0.129). In terms of NIHSS, except for one patient with a score of 0, all patients had a score of 1 in the isolated dysarthria group. In the prominent group, 14 (48.3%) patients had a score of 2, five patients (17.2%) had a score of 3, and four patients (13.8%) had a score of 1. As for the TOAST classification in DWI positivity, the percentage of small vessel disease was the highest in both groups (*n* = 8, 66.7% vs. *n* = 15, 51.7%).

## 4. Discussion

In the present study, we investigated the clinical characteristics, neuroimaging findings, and diagnoses of patients with acute isolated or prominent dysarthria visiting the ED.

Acute dysarthria is a common neurological symptom encountered in the ED and occurs in various neurological and medical conditions. Interestingly, acute dysarthria rarely occurs alone or is a major symptom. In acute ischemic stroke, acute dysarthria is often accompanied by limb weakness and rarely occurs alone or with minor neurological symptoms without motor weakness [7,8,10,13]. Therefore, there has been little clinical interest in acute dysarthria, and previous studies have shown limited data on acute stroke. Moreover, the definition of acutely isolated dysarthria and the criteria of enrolled patients were described heterogeneously in each study. 

In clinical practice, it is natural for emergency physicians to first consider ischemic stroke, which has a golden time for treating patients with acute dysarthria. In the present study, acute ischemic stroke accounted for most causes at 69.3%, supporting the evidence that clinicians should pay attention to the causes of stroke. However, hemorrhagic stroke was the cause in only one patient, presumably because symptoms tended to be more severe and complicated in patients with hemorrhagic stroke visiting the ED. Even though we reviewed the data thoroughly, only one case of isolated dysarthria in hemorrhagic stroke was identified [10]. However, since 30.7% of dysarthria cases in the present study were caused by other non-stroke causes, clinicians should consider that acute dysarthria in the ED may also be caused by neurological and medical causes other than stroke. Neurological diseases, such as brain tumor, Parkinson’s disease, multiple sclerosis, seizure, and multiple cranial neuropathies, as well as metabolic causes, toxic causes, aortic dissection, infectious causes, and psychiatric disorders, can cause acute dysarthria; therefore, a comprehensive approach is needed.

In terms of additional symptoms, the common symptoms in the stroke group were facial palsy (13.5%) and gait disturbance or imbalance (15.4%). Similar to our results, Urban [7] and Kim [9] reported that facial palsy was commonly accompanied by 58.8% and 69.2%. In our DWI findings, we confirmed that the lesion involved the corticobulbar fiber (corona radiata, internal capsule, and subcortex) in all cases of facial palsy. Although the authors cannot clearly explain why dysarthria is often accompanied by gait disturbance, we were able to confirm that patients with gait disturbance or imbalance had lesions in the thalamus, brainstem, cerebellum, and frontal lobe on DWI findings [16].

Generally, it is reasonable to assume that patients with mild neurological symptoms may have small lesions on magnetic resonance imaging. The isolated dysarthria group had a higher rate of TIA, single and small lesions, and small-vessel occlusion in TOAST than the prominent dysarthria group, albeit without reaching statistical significance, owing to the small sample size. However, we need to pay attention clinically to the finding that even mild isolated symptoms can be caused by multiple or territorial lesions or large vessel occlusion [7].

The strength of our study is that the patients’ neurological evaluation was performed continuously and consistently by a single neuro-emergency expert in the ED, and both NIHSS and TOAST were evaluated, recorded, and analyzed. On the other hand, this study has limitations in that it had a retrospective design, the sample size was small, and it did not include all emergency center patients.

## 5. Conclusions

Acute isolated or prominent dysarthria in the ED mostly presented as clinical symptoms of AIS, but other non-stroke and medical causes were not uncommon. In acute dysarthria with ischemic stroke, multiple territorial lesions are considered a cause, in addition to small and single lesions.

## Figures and Tables

**Table 1:** Characteristics of patients with isolated or prominent dysarthria.

Variables	Isolated Dysarthria	Prominent Dysarthria	*p*-Value
	(*n* = 28)	(*n* = 47)	
Age, years	70.0 (58.0–79.0)	71.0 (60.0–80.0)	0.793
Age group, years *			
40–59	7 (25.0)	12 (25.5)	0.836
60–79	14 (50.0)	21 (44.7)	
≥80	7 (25.0)	14 (29.8)	
Male	16 (57.1)	28 (59.6)	0.713
Onset-to-door time, min	689.0 (87.0–1350.0)	1283.0 (411.0–4020.0)	0.042
Coexisting condition			
Hypertension	20 (71.4)	26 (55.3)	0.166
Diabetes mellitus	9 (32.1)	14 (29.8)	0.831
Dyslipidemia	5 (17.9)	9 (19.1)	0.890
Current smoker	4 (14.3)	3 (6.4)	0.413
Atrial fibrillation	0 (0.0)	2 (4.3)	0.526
Ischemic stroke	1 (3.6)	1 (2.1)	0.707
Coronary artery disease	0 (0.0)	3 (6.4)	0.289
Chronic renal disease	2 (7.1)	3 (6.4)	0.898
Antithrombotic agent			
Aspirin	3 (10.7)	10 (21.3)	0.348
Clopidogrel	3 (10.7)	2 (4.3)	0.356
Rivaroxaban	0 (0.0)	1 (2.1)	0.437

Values are expressed as medians (interquartile ranges) or numbers (%). * The sum of the proportions is not equal to 100%, owing to rounding.

**Table 2:** Final diagnosis of patients with isolated or prominent dysarthria.

Variables	Total	Isolated Dysarthria	Prominent Dysarthria
	(*n* = 75)	(*n* = 28)	(*n* = 47)
Stroke	52 (69.3)	18 (64.3)	34 (72.3)
AIS	37 (49.3)	11 (39.3)	26 (55.3)
TIA	14 (18.7)	7 (25.0)	7 (14.9)
ICH	1 (1.3)	0 (0.0)	1 (2.1)
Non-stroke	23 (30.7) *	10 (35.7) *	13 (27.7) *
Metabolic	7 (9.3)	3 (10.7)	4 (8.5)
Toxic	6 (8.0)	4 (14.3)	2 (4.3)
Brain tumor	2 (2.7)	1 (3.6)	1 (2.1)
Parkinson disease	2 (2.7)	1 (3.6)	1 (2.1)
Multiple sclerosis	1 (1.3)	0 (0.0)	1 (2.1)
Seizure	1 (1.3)	1 (3.6)	0 (0.0)
Multiple cranial neuropathy	1 (1.3)	0 (0.0)	1 (2.1)
Aortic dissection	1 (1.3)	0 (0.0)	1 (2.1)
Appendicitis	1 (1.3)	0 (0.0)	1 (2.1)
Psychiatric	1 (1.3)	0 (0.0)	1 (2.1)

Values are expressed as numbers (%). * The sum of the proportions is not equal to 100%, owing to rounding. AIS, acute ischemic stroke; TIA, transient ischemic attack; ICH, intracerebral hemorrhage.

**Table 3:** Various additional symptoms in patients with prominent dysarthria.

Additional Neurologic Symptoms	Total *	Non-Stoke *	Stroke *
	(*n* = 47)	(*n* = 13)	(*n* = 34)
Gait disturbance/imbalance	9 (19.2)	1 (7.7)	8 (23.5)
Facial palsy	8 (17.0)	1 (7.7)	7 (20.6)
Mild hemiparesis	5 (10.6)	2 (15.4)	3 (8.8)
Dizziness	4 (8.5)	3 (23.1)	1 (2.9)
Limb ataxia	3 (6.4)	0 (0.0)	3 (8.8)
Sensory change	3 (6.4)	0 (0.0)	3 (8.8)
Transient motor weakness	3 (6.4)	0 (0.0)	3 (8.8)
Swallowing difficulty	2 (4.3)	1 (7.7)	1 (2.9)
Acute memory loss	1 (2.1)	1 (7.7)	0 (0.0)
Chest pain	1 (2.1)	1 (7.7)	0 (0.0)
Diplopia	1 (2.1)	0 (0.0)	1 (2.9)
Fever	1 (2.1)	1 (7.7)	0 (0.0)
Headache	1 (2.1)	0 (0.0)	1 (2.9)
Hip pain	1 (2.1)	0 (0.0)	1 (2.9)
Leg weakness	1 (2.1)	0 (0.0)	1 (2.9)
Drowsy mentality	1 (2.1)	1 (7.7)	0 (0.0)
Tongue palsy	1 (2.1)	1 (7.7)	0 (0.0)
Blurred vision	1 (2.1)	0 (0.0)	1 (2.9)

Values are expressed as numbers (%). * The sum of the proportions is not equal to 100%, owing to rounding.

**Table 4:** Comparison between isolated and prominent dysarthria in patients with acute ischemic stroke or TIA.

Variables	Isolated Dysarthria	Prominent Dysarthria	*p*-Value
	(*n* = 18)	(*n* = 33)	
Clinical diagnosis			0.204
Ischemic stroke	11 (61.1)	26 (78.8)	
TIA	7 (38.9)	7 (21.2)	
DWI positivity	12 (66.7)	29 (87.9)	0.154
Single and small lesion	10 (83.3)	16 (55.2)	
Multiple or territorial lesions	2 (16.7)	13 (44.8)	
NIHSS at admission to ED in DWI positivity			<0.001
0	2 (16.7)	3 (10.3)	
1	11 (83.3)	4 (13.8)	
2	0 (0.0)	14 (48.3)	
3	0 (0.0)	5 (17.2)	
4	0 (0.0)	3 (10.3)	
TOAST in DWI positivity			0.566
1	3 (25.0)	7 (24.1)	
2	8 (66.7)	15 (51.7)	
3	0 (0.0)	4 (13.8)	
4	0 (0.0)	0 (0.0)	
5	1 (8.3)	3 (10.3)	

Values are expressed as numbers (%). DWI, diffusion-weighted imaging; ED, emergency department; NIHSS, National Institutes of Health Stroke Scale; TIA, transient ischemic attack; TOAST, Trial of Org 101072 in Acute Stroke Treatment.

## Data Availability

Not applicable.
